# Distributed Impact Wave Detection in Steel I-Beam with a Weak Fiber Bragg Gratings Array

**DOI:** 10.3390/s23042194

**Published:** 2023-02-15

**Authors:** Yuan Wang, Neil A. Hoult, Joshua E. Woods, Hannah Kassenaar, Xiaoyi Bao

**Affiliations:** 1 School of Electrical Engineering and Computer Science, University of Ottawa, Ottawa, ON K1N 6N5, Canada; 2 Department of Civil Engineering, Queen’s University, Kingston, ON K7L 3N6, Canada; 3 Department of Physics, University of Ottawa, 25 Templeton Street, Ottawa, ON K1N 6N5, Canada

**Keywords:** structural health monitoring, distributed sensor, impact wave detection

## Abstract

In this paper, acoustic, dynamic and static strain variations along a steel I-beam generated by an impact load are reconstructed simultaneously within a single measurement. Based on the chirped pulse φ-OTDR system with the single-shot measurement technique, both a higher strain-sensing resolution and a higher measurable vibration frequency are achieved. In addition, a weak fiber Bragg gratings array (WFBGA) with enhanced Rayleigh reflection is employed as a sensor, providing high signal-to-noise ratio Rayleigh traces, resulting in lower measurement uncertainty. In the experiments, the damping constant and fundamental frequency of the damped harmonic oscillator could then be measured based on the recovered strain variation profile for further structural health analysis. Compared with commercial strain gauges, linear potentiometers, and OFDR systems, the proposed sensing system ensures a distributed, quantitative, and high-frequency sensing ability, with an extensive range of potential applications.

## 1. Introduction

Infrastructure assets, such as buildings, roads, and highways, are critical to the day-to-day lives of people around the world. However, these structures have an expected service life and deteriorate due to the aging of materials, overloading, lack of sufficient maintenance, and extreme events [1]. To address this challenge, a real-time distributed monitoring system would help engineers and managers assess the condition of infrastructure assets and repair them in a timely and safe fashion. Benefiting from their geometric (small size, sensor length, flexibility, and lightweight) and metrological advantages (accuracy, high recording frequency, millimetric spatial resolution, and sensitivity) [2], distributed optical fiber sensors (DOFS) have been widely used in civil engineering as a tool to assess the health and monitor infrastructure condition for the oil and gas industry [3,4], the power transmission industry [5,6], structural monitoring [7,8], the transportation industry [9,10], and security monitoring [11,12]. In addition, DOFS have been the subject of numerous studies related to the structural health monitoring of civil engineering structures, including investigating their ability to measure strains in reinforced concrete beams [8], detect internal cracks in concrete structures [13,14], and the stress transfer mechanisms between the components of optical fiber sensors [15]. However, challenges arise when strain changes are small due to localized deterioration or rapidly change with time due to extreme loading, represented by the requirement for high accuracy of the order of microstrain for static strain and measurement of peak strains that change at the Hz or even kHz rate. Both conditions are difficult to measure accurately due to noisy data [16], especially when the structural strain change under different loading is in the range of a few microstrains. This is because the strain reading requires phase demodulation, in which phase unwrapping varies with the frequency drift of the laser wavelength. While dynamic strain measures a time-dependent relative change in intensity, the intensity-based demodulation method misses the quantitative change needed for the strain reading recovery [17].

Moreover, as the need for lower embodied carbon in civil engineering structures rises, there will be an increasing demand to achieve more complex structural forms, such as those with novel materials or optimized geometry, for which the behavior of these elements may need to be studied in the lab and field, including under high-frequency loading. This paper investigates different techniques to capture distributed dynamic (from a few Hz to 1 kHz) and small static (<1 με) strains.

With this specific dynamic strain requirement of high-speed response and the requirements for static strain accuracy, the best candidate is a phase-sensitive optical time-domain reflectometer (φ-OTDR) which is capable of measuring dynamic strain with a wide frequency response and a high strain accuracy [17,18]. At the same time, optical frequency-domain reflectometry (OFDR) has been shown to be suitable for measuring static strain with a high spatial resolution at a low sampling rate [19]. However, the phase demodulation process in these sensing systems relies on the beating between the backscattered light and the original laser output, which is actually a Mach–Zehnder interferometer that possesses a high sensitivity to environmental noises, such as temperature changes and vibrations. Recently, a novel chirped pulse φ-OTDR (CP φ-OTDR) sensing system using a distributed feedback (DFB) laser without an optical interferometer-based phase demodulation scheme has been proposed [20]. The external disturbance-induced optical path length changes are translated into local time delays within the time window of reflected Rayleigh traces. The direct single-shot time delay measurement without coherent detection offers a capability of real-time high-accuracy strain, and high-frequency measurements (only limited by fiber length) [21], achieved by using a low-cost DFB laser (1 MHz), where no environmental dependence in the demodulation process is observed because of the use of direct detection in the electrical domain. In addition, the weak fiber Bragg gratings array (WFBGA) is used as a sensor in which the combination of the FBGs and Fabry–Pérot interferometer (FPI) between gratings allows for a distributed measurement and the delay spectrum to provide a higher strain accuracy with a single pulse measurement, critical for impact wave monitoring.

In this paper, the proposed CP φ-OTDR is used for the first time in a civil engineering application to measure the impact load response of a steel I-beam, including the acoustic wave, damped harmonic oscillations, and static strain measurement. One challenge with this measurement is the high-frequency acoustic wave. For the OFDR technique, the frequency sweep process lowers the highest sampling rate below 100 Hz, and the high-frequency components are difficult to capture. However, the proposed sensor in this paper uses a chirped pulse for tensile/compressive strain measurement with a single-shot measurement, which enables a higher sampling rate that is only limited by the sensor length. Another challenge is that the environmental noise and generated sound waves make coherent detection invalid, resulting in no readout data for the acoustic wave detection. However, the proposed sensor directly measures the time delays between selected windows for the same location in time-domain traces, without using the phase demodulation process. The specific objectives of this paper are to: (1) measure the dynamic response of the steel beam, including the acoustic wave and low-frequency dynamic strain variations, (2) compare the static strain measurement from WFBGA-based φ-OTDR to other traditional strain measurement technologies, including a commercially available OFDR-based DOFS system as well as a traditional electrical resistance strain gauge, and (3) investigate the damping parameters in the low-frequency dynamic strain section and the impact of the drop height on the response of the steel I-beam.

## 2. Theoretical Analysis and Experimental Setup

**Figure 1:**
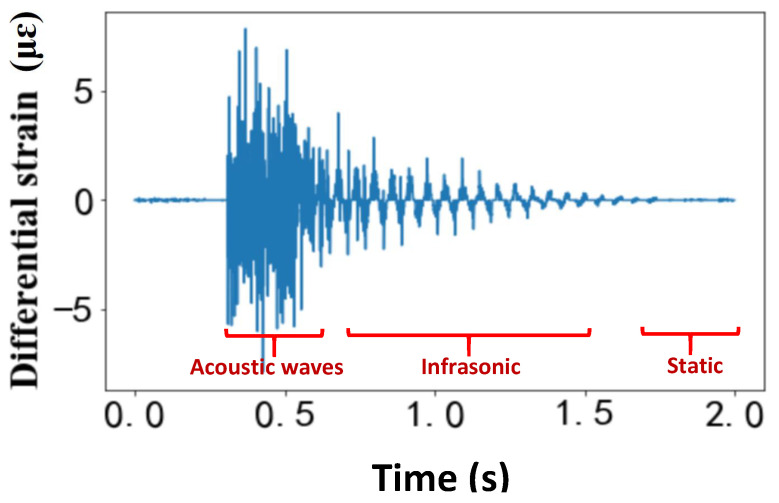
Expected differential strain variations of the steel I-beam due to the impact load.

In this study, a lumped mass was released from a desired height, which generated an impact force on the top flange of a simply supported beam at midspan. Figure 1 shows the differential strain variations of the steel beam structure after the impact force was applied. At the very beginning, surface acoustic waves are initially generated and propagate along the length of the beam. The frequency range of the generated surface acoustic wave is determined by the kinetic energy of the lumped mass. In the second stage, the beam structure experiences a damped free-vibration response, in which the beam exhibits harmonic oscillations. Because the high-frequency acoustic wave has a large attenuation, it vanishes quickly. The frequency of the damped harmonic oscillations is usually in the infrasonic range and depends on the parameters of the beam (e.g., length and stiffness). Finally, as the beam comes to rest with the additional applied mass, a static strain is expected when the beam stops oscillating. To quantify the beam behavior, these three states of the beam response need to be monitored and quantified, which can be challenging when only using one sensor.

Consider that the damped harmonic oscillator differential equation to describe the “infrasonic” section of the response is given by:

(1) $$\ddot{x}+2\gamma \dot{x}+\omega_{0}^{2}x=\delta \left(t\right)$$

where *x* is the vertical displacement of the beam structure, γ is the intrinsic damping parameter, and $\omega_{0}$ is the fundamental frequency for the system. This equation describes the impulse δ(t) response, it can be solved for the initial condition x(t)=0, and the solution is given by:

(2) $$x\left(t\right)=\Theta \left(t\right)\frac{\sin\left[\sqrt{\omega_{0}^{2}-\gamma^{2}}t\right]}{\sqrt{\omega_{0}^{2}-\gamma^{2}}}e^{-\gamma t}$$

where Θ(t) is the step function or Heaviside function. By fitting the measured strain data with different impact weights, these two parameters can be determined for structural health monitoring and used to understand the condition of the structure. It should be noted that these two parameters do not depend heavily on the falling weight (or drop height) in these experiments. After the low-frequency oscillations, the beam structure will come to rest with the lumped mass on top. In this state of equilibrium, the beam experiences bending stresses with compression above the neutral axis and tension below.

**Figure 2:**
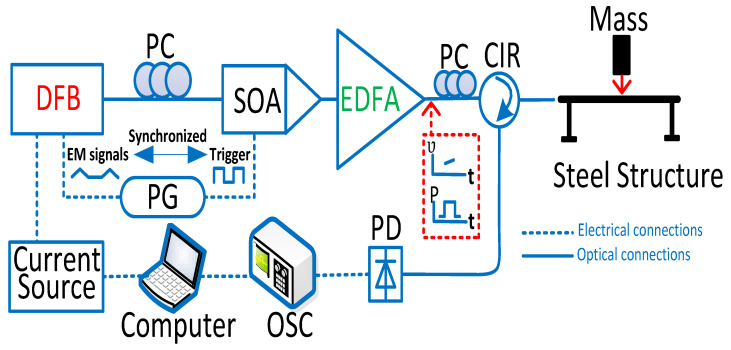
Schematic of the chirped pulse φ-OTDR sensing system. DFB, distributed feedback laser; SOA, semiconductor optical amplifier; EDFA, Erbium-doped fiber amplifier; PC, polarization controller; PG, pulse generator; PD photo-detector; OSC, oscilloscope; EM, electrical modulation; CIR, circulator.

Figure 2 depicts the chirped pulse φ-OTDR system used for the distributed impact wave measurements. The key components included: the chirped pulse generator scheme which is composed of a DFB laser diode (CQF938/500, JDS Uniphase), a pulse generator (PG) (8130A, Hewlett Packard) and a semiconductor optical amplifier (SOA) (OPB-10-10-N-C-FA, Kamelian) driven by an electrical circuit. A polarization controller was utilized to vary the state of polarization of the output light from the DFB laser so as to get a maximum efficiency from the SOA. By applying an electrical triangle signal, which was generated by a pulse generator, the output frequency of the DFB laser experienced a continuous variation. Meanwhile, a synchronized trigger signal actuated the SOA, yielding an optical chirped pulse with a pulse width of 8 ns and a linear frequency chirping range of 1.25 GHz. After amplifying with a Erbium-doped fiber amplifier (EDFA), the chirped pulse signal was sent to the weak FBG array. The backscattered Rayleigh signal was transferred from optical signals to electrical signals via a 1G bandwidth photodetector (PD) and then was collected in real time by a digital oscilloscope (DSO81204B, Agilent) with a sampling rate of 40 GSa/s.

**Figure 3:**
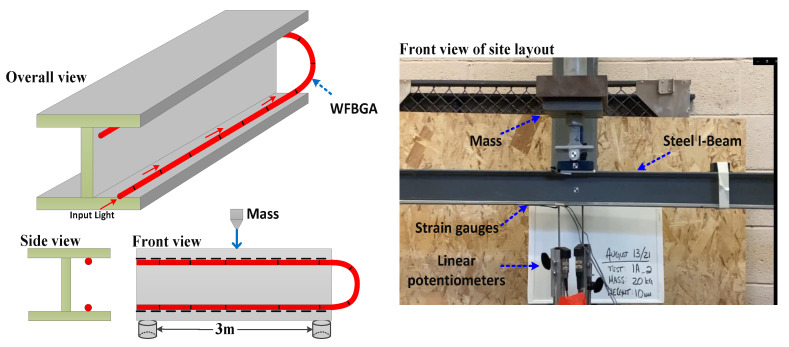
Layout of the weak FBG array on the steel I-beam.

Figure 3 shows the impact wave generation and installation of the FBG array on the surface of the steel I-beam. The length of the I-beam was about 3 m, and the total length of our WFBGA sensor was about 6 m. The WFBGA had an enhanced reflectivity of −30 dB and a grating period of 10 cm. The WFBGA was manufactured by the fiber sensing lab at Wuhan University of Technology. The array was glued to the beam from the top of the bottom flange to the bottom of the top flange to measure the tension and compression behavior. Two strain gauges and two linear potentiometers (LPs) were also used to monitor the generated impact waves. The strain gauges were mounted approximately 160 mm from the center of the beam, while the 2 LPs were mounted at the midspan of the beam to measure the displacement on either side of the flange. Because a single load was applied in the center of a simply supported beam in the drop test, it was anticipated that the strain would decrease linearly towards the supports. Thus, a linear interpolation was appropriate to compare strain values measured at different locations along the length of the beam.

## 3. Experimental Results

To evaluate the capability of the proposed sensing system to measure the dynamic response of the beam, a series of tests were conducted with two loading combinations. A 20 kg mass was dropped onto the beam for each test using an impact mechanism. The impact mechanism consisted of a steel wire and pulley system that was used to raise the mass to 10 mm or 40 mm above the top of the beam. The acquisition rate of the WFBGA-based CP φ-OTDR was set as 2 or 4 kHz, and the spatial resolution was 0.8 or 1 m. The sampling rate of the strain gauges and OFDR systems were 2 kHz and 100 Hz, respectively.

### 3.1. Static Strain Measurement

**Figure 4:**
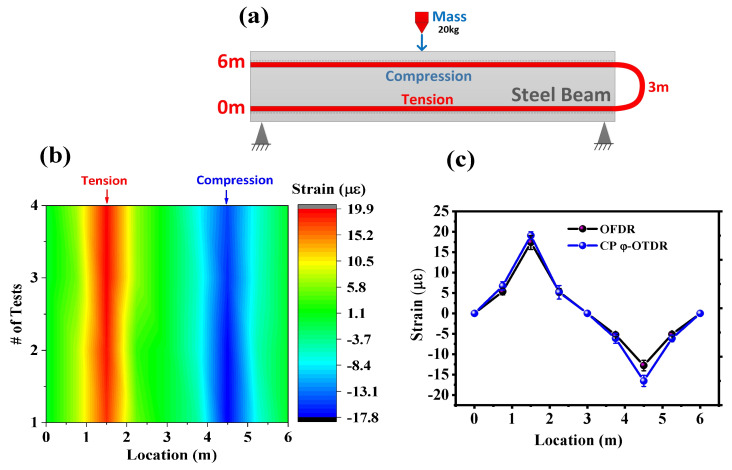
(**a**) layout for static strain measurement; (**b**) distributed static strain monitoring along the steel I−beam based on a chirped pulse φ−OTDR; (**c**) static strain distribution from OFDR and chirped pulse φ−OTDR system (1 m spatial resolution).

Before assessing the dynamic performance of the strain-sensing technologies, the ability of the two distributed fiber-optic sensing technologies to measure the static strain response was assessed in the third stage of the impact response as shown in Figure 1. The layout of the fiber-optic cable installed on the beam is shown in Figure 4a. The WFBGA was glued using epoxy to the inside of the top flange loops around to the bottom left flange of the beam. The total length of the test fiber was about 10 m, of which only a 6 m section was bonded to the test beam. By sending a chirped pulse as the interrogation signal, the reflected Rayleigh traces from the weak FBG array could be continuously collected. Since the local differential strain variation was translated into local time delays between two single-shot-measured Rayleigh traces, the strain at any given time was the integration of the differential strain variations up to that point in time. The minimum detectable strain variation (sensitivity) was finally determined by the sampling rate (time-delay resolution) and frequency chirping rate of the pulse. Other measurement errors of the laser frequency drifting noise could be solved by using the demodulation results of the nondisturbed section to compensate the laser frequency noise.

Note that the first stage in Figure 1 contained high-frequency components beyond the maximum measurable frequency of the system, which resulted in a larger measurement uncertainty for the strain variation demodulation in the “static” part. Thus, to avoid the impact of the high-frequency acoustic waves, the first reference trace to calculate the strain variation profile of the “infrasonic” and “static” parts was set as the trace from the beginning of the test, in which there was no strain applied. Figure 4b shows the static strain distribution for the steel I-beam, in which the first section would experience tension and the fiber on the bottom of the top flange experienced compressive strains. In the experiments, we repeated the measurement four times, and the y-axis of Figure 4b represents the serial number of tests. It was noted that the spatial resolution of OFDR needed to be modified to enable a direct comparison since the gauge length and longitudinal locations of the fiber on the beam cross-section were different. With the assumption of a linear strain variation along the I-beam from the support ends to the midspan, the theoretical strain value at a different location along the beam could be calculated based on the strain gauge readings. Thus, the strain variation profile with different spatial resolutions could be obtained by integrating these values within the spatial resolution range. The static strain measurement comparison between the OFDR system and CP φ-OTDR is shown in Figure 4c, which shows a good consistency within 2 με variation between results. The measurement uncertainty from different tests is shown in the error bar in Figure 4c with an average value of about 1.2 με.

It was noted that there was a strain mismatch near the load points (the supports and where the mass was dropped). As a result, when the actual strain was integrated over the middle 1 m, the compressive strain was less than the tensile strain. The results showed that the tension strain at the top midspan (1.5 m) were slightly higher than the compressive strain results at the bottom midspan (4.5 m).

### 3.2. Dynamic Strain Measurement

**Figure 5:**
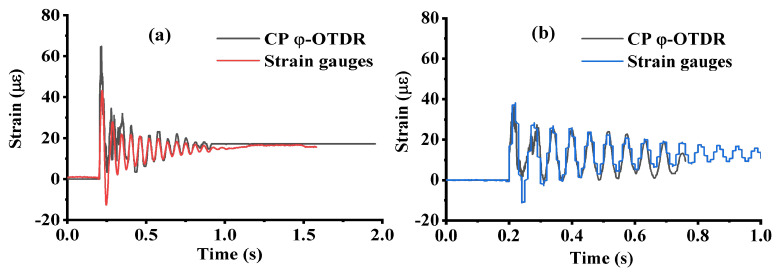
Strain–time response comparison between a CP φ−OTDR and strain gauge at midspan with different spatial resolutions of (**a**) 0.8 m and (**b**) 1 m.

For the dynamic measurements, the lumped mass was released from a height of 40 mm or 10 mm, depending on the test, on the midspan of the beam. A horizontal cylindrical rod was placed on the bottom of the impact mechanism and perpendicular to the longitudinal axis of the beam to simulate a point load. To prevent the beam from sliding or bouncing during the impact tests, a ratchet strap was placed around the beam at the outer end of each support to not interfere with the behavior of the beam. The impact load response of the beam structure with a drop height of 10 mm was monitored by the sensor array and strain gauges, and the results are shown in Figure 5. As was discussed for the results in Figure 1, the beam underwent three distinct states, including a high-frequency vibration, damped oscillation and static strain, which were captured differently by these two types of sensors. Apart from the difference in distributed and point sensors, they also showed a different capacity for high-frequency vibration detection.

In Figure 5, the CP φ-OTDR results are compared against the strain gauges results, which were adjusted to have a 1000 mm or 800 mm gauge length. For the damped oscillation and static portions of the response, the CP φ-OTDR measurements and the modified strain gauge results were in good visual agreement, while there was some noise in the CP φ-OTDR measurements in the first part of the response (from approximately t = 0.2 s–0.3 s). The noise was due to high-frequency vibrations in the beam caused by the impact (which corresponded to the sound of the mass impacting the beam). The sound wave coupled with the WFBGA and generated a high-frequency strain variation with a comparable strain amplitude to the minimum detectable value (approximately nε) that decayed rapidly. However, this sound wave was not detected by the electrical strain gauge due to the limited sensitivity and lower coupling efficiency compared with the WFBGA. After that, the mass, together with the beam, entered the damped oscillation stage after the first 0.1 s after impact, which could be captured by both CP φ-OTDR and strain gauges.

**Figure 6:**
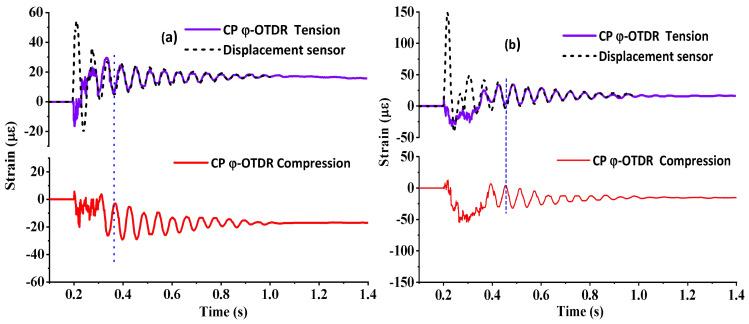
Strain–time response comparison between the CP φ−OTDR and linear potentiometer (LP) at midspan with different drop heights of (**a**) 10 mm and (**b**) 40 mm.

The impact-force-induced negative strain (compression) and positive strain (tension) could be calculated by the vertical midspan displacement of the beam, which was measured by the linear potentiometer (LP) sensor and the displacement–tensile strain coefficient. The displacement–tensile strain coefficient was calculating using the beam properties and the integration length, which was 0.8 m for the results in Figure 6. The dashed line in Figure 6a shows the equivalent strain value based on the displacement sensor by using the displacement–tensile strain coefficient of 35 με/mm, while the solid line shows the results from the CP φ-OTDR. It was noted that local stress concentrations in the vicinity of the point loads introduced a discrepancy between the peak tensile and compressive strain, which meant that the displacement–strain coefficient was different for tensile and compressive strains. Here, we only compared the tensile strain results from the CP φ-OTDR and LP sensor to verify the measurement accuracy of the proposed system. The result from the two sensors was largely in good agreement, with no variation of more than 2 με after the sound vibration induced the high-frequency vibration section (about 0.2 s–0.3 s). To verify the displacement–strain coefficient, the mass drop height was set as 40 mm, and the impact wave generation process can be found in the video in the Supplementary Materials. The dynamic strain response was obtained by applying the displacement–strain coefficient to convert the displacement to the strain, which is shown in Figure 6b. The two tests with different drop heights showed a different initial impact behavior, with the impact of the acoustic wave (noisy profile at the beginning) lasting for a longer time for the 40 mm drop, which was to be expected since more energy was imparted to the beam. For both tests, the vertical dashed line showed that the bottom and top flange had symmetrical responses. More importantly, the dynamic strain measurement from the CP φ-OTDR showed a good consistency with the result from the LP sensor, except for the acoustic wave detection at the beginning of the test.

Another interesting point that should be noted is the time duration of the high-frequency section was doubled from about 0.1 s to 0.2 s, since the 40 mm height mass created a larger impact force that led to stronger sound waves and more energy transfer from potential energy to kinetic energy, which led to a longer duration time of the decay process for the high-frequency acoustic wave. The decay process was related to the γ parameter and the maximum amplitude of the displacement.

**Figure 7:**
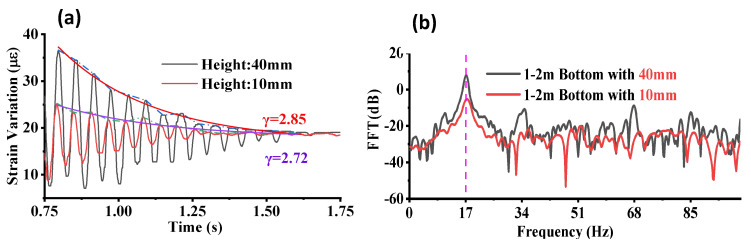
(**a**) The intrinsic damping parameter fitted in the damped oscillation section and (**b**) the natural frequency with different drop heights.

By selecting the damped harmonics oscillation section of the response, the intrinsic damping parameter was fitted to the peak value of each vibration period, as shown in Figure 7a. Similar to what was predicted in the theoretical analysis based on Equation (1), the damping parameter did not depend significantly on the drop height (or impulse force). Thus, the different drop heights yielded a similar result of 2.85 and 2.72 for the intrinsic damping parameter. The small difference may have come from the system noise and fitting errors. In addition to the damping parameter, the fundamental frequency of the system was evaluated from the fast Fourier transform (FFT) analysis, as shown in Figure 7b. A dominant peak could be found at the same location of 17 Hz in both measurements with different drop heights. With a larger impulse force from the 40 mm drop test as shown in Figure 7b, higher harmonic components were excited. The high potential energy led to a higher kinetic energy with a higher impact wave frequency, as illustrated in Figure 7b.

**Figure 8:**
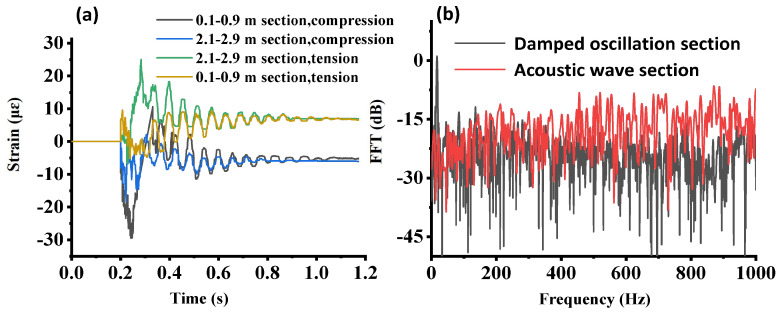
(**a**) Distributed dynamic strain measurement by the CP φ−OTDR for the left span and right span, and (**b**) FFT analysis of the damped oscillation section and acoustic wave section with the same drop height of 40 mm at midspan.

In the previous discussion, the dynamic measurements taken/averaged about the midspan of the beam were investigated. It is also important to monitor the dynamic strain distribution along the beam, as damage may occur at other locations. Figure 8a presents the strain/compression for the left and right spans. In the 0.1–0.9 m range, the CP φ-OTDR provided a higher compression measurement than that in the 2.1–2.9 m range in the first 0.1 s after impact, which was an unexpected result from a mechanical strain point of view. The reason was that the differential strain variation between two adjacent single-shot measurements for the two side-spans was supposed to be a few nanostrains. However, for the real measurement, this differential strain could be up to the sub-microstrain range and with the same sign due to the broadband frequency of the acoustic waves exceeding the sampling rate of the proposed system (2 kHz). Thus, the overall strain variations showed an irregular strain profile at the beginning when integrating the differential strains over time. To solve this problem and give a more precise strain variation profile at the beginning of the test, further improvements in sensing accuracy and sampling rate are required.

Finally, the FFT analysis of the acoustic wave section is shown in Figure 8b. It shows that the broadband acoustic frequency range in the acoustic wave section was excited compared with the damped oscillation section, which only had a dominant peak at the fundamental frequency location. The mass, together with the I-beam, vibrated based on the assumption of a rigid body in which the first modal frequency was dominant. On the other hand, the lumped mass with a higher drop height had a larger kinetic energy and excited more high-frequency components at the beginning of the test. When the drop height was increased, the kinetic energy could not be fully absorbed by the I-beam. The nonabsorbed kinetic energy led to different oscillations of the I-beam in a very short time at a higher frequency. It was noted that this broadband high-frequency acoustic wave detection was only enabled by the proposed system, which could reveal potential cracks introduced by the external load. The diagnosis of structural damage or cracks based on the FFT analysis of generated acoustic waves illustrated that the rigid body assumption should be used carefully for a high-frequency impact condition.

## 4. Conclusions

This paper demonstrated a distributed multiparameter sensor for acoustic wave, dynamic strain, and static strain sensing using a weak FBG array fiber. A proof-of-concept impact test was conducted by dropping a known mass from a fixed height onto a simply supported beam. The detection of the distributed impact response required three parameters in one measurement: (1) sound wave response; (2) vibration frequency; and (3) small dynamic strain. This was very challenging, which is exactly the novel point of this research. What makes the electronic time-delay demodulation of the chirped pulse OTDR stand out is that the electric delay measurement is not an optical interferometer, which is insensitive to the sound impact. Hence, this I-beam subjected to impact loading was a good example to demonstrate the advantage of the electric time-delay correlation for the chirped pulse OTDR response from a weak FBGs array with a single pulse measurement without coherent detection, which had never been demonstrated before. The time-dependent impact response, including the broadband frequency vibration of the I-beam, was successfully detected by the CP φ-OTDR system with a high sampling rate up to 2 kHz in a distributed manner. In the experiments, the sampling rate was limited by the memory size of the oscilloscope. In addition, the results from the CP φ-OTDR system were compared with a commercially available OFDR system (only static strain), strain gauges, and a linear potentiometer, showing a good agreement with errors below 3 με. The proposed sensor showed the capacity to detect high-frequency acoustic waves, while the OFDR had no readout for the beginning section of the impact response, which was not captured by the strain gauges and LPs, as they are point sensors, due to the small coupling coefficient and limited sensitivity.

## Data Availability

The data presented in this study are available upon request from the corresponding author.

## Supplementary Materials

The following supporting information can be downloaded at: https://www.mdpi.com/article/10.3390/s23042194/s1, Video S1: Impact wave generation with drop height of 40 mm.
